# Feeding requirements of white sharks may be higher than originally thought

**DOI:** 10.1038/srep01471

**Published:** 2013-03-18

**Authors:** J. M. Semmens, N. L. Payne, C. Huveneers, D. W. Sims, B. D. Bruce

**Affiliations:** 1 Fisheries, Aquaculture and Coasts Centre, Institute for Marine and Antarctic Studies, University of Tasmania, Hobart 7001, Australia; 2 School of Biological, Earth and Environmental Sciences, University of New South Wales, New South Wales 2052, Australia; 3 Threatened, Endangered, and Protected Species subprogram, SARDI – Aquatic Sciences, South Australia 5024, Australia; 4 School of Biological Sciences, Flinders University, South Australia 5042, Australia; 5 Marine Biological Association of the United Kingdom, The Laboratory, Citadel Hill, Plymouth PL1 2PB, UK; 6 Ocean and Earth Science, National Oceanography Centre Southampton, University of Southampton, Waterfront Campus, Southampton SO14 3ZH, UK; 7 Centre for Biological Sciences, Building 85, University of Southampton, Highfield Campus, Southampton SO17 1BJ, UK; 8 Wealth from Oceans Flagship, CSIRO Marine & Atmospheric Research, Hobart 7001, Australia

## Abstract

Quantifying the energy requirements of animals in nature is critical for understanding physiological, behavioural, and ecosystem ecology; however, for difficult-to-study species such as large sharks, prey intake rates are largely unknown. Here, we use metabolic rates derived from swimming speed estimates to suggest that feeding requirements of the world's largest predatory fish, the white shark (*Carcharodon carcharias*), are several times higher than previously proposed. Further, our estimates of feeding frequency identify a clear benefit in seasonal selection of pinniped colonies - a white shark foraging strategy seen across much of their range.

Understanding the energetic requirements of organisms in their natural environment is fundamental to ecosystem ecology, as the energetic benefits and costs associated with their activities will heavily influence life-history strategies and trophic relationships. Inherent difficulties in studying marine predatory behaviour in the wild have hindered our understanding of the energetic requirements and associated trophic relationships of apex predators. In the case of pelagic predatory sharks, approaches that provide energetic data are urgently needed, as many of these species are highly vulnerable to overexploitation^1^.

White sharks *Carcharodon carcharias* (Lamnidae) are apex marine predators with a circumglobal distribution. Their longevity, late maturity and low fecundity renders them highly susceptible to overexploitation^2^. The population status of white sharks is poorly known over the species' range due to a lack of robust abundance indicators, given it is protected throughout much of its range and only caught as a fisheries bycatch species or as part of shark control programs^2^. Additionally, despite their protected status, white sharks are still regularly incidentally caught in various fishing gear throughout their range^3^,^4^. Even at very low levels of anthropogenic mortality, modelled white shark populations have greatly increased doubling times^5^, and declines in relative catch rates have been reported in parts of their range, e.g. Refs. 3,6. There is however, conjecture surrounding the magnitude of some of these declines^7^,^8^,^9^ and some evidence for slight increases in relative catch rates in the last 10–20 years in parts of their range, e.g. Refs 3,4.

Shifting from a predominantly piscivorous diet to one dominated by marine mammals at approximately 3.4 m in total length^10^, large white sharks are regular visitors to seal breeding colonies. For example, the Neptune Islands (South Australia) supports the largest seal colony in Australia, and white sharks are most abundant in the area during winter-spring when weaned New Zealand (NZ) fur seals *Arctocephalus forsteri* are present^11^.

Energy requirements of large sharks are poorly documented. The only published study of white shark energetics in the wild estimated the field metabolic rate (MR) of a single individual from telemetered muscle temperature data as the individual moved from cold to warm water^12^. The authors used their MR estimates to suggest a 943 kg white shark could survive on 30 kg of marine mammal blubber for approximately 1.5 months; a widely cited figure that has perpetuated the assumption that large sharks only need to feed every few weeks to maintain net energy gain.

Here, we combine estimates of swimming speeds [Fig. 1] and measurements of standard (resting for an obligate ram-ventilator) MR (SMR) in young-of-the-year (YOY) white sharks^13^, with swim-tunnel respirometry data from closely-related shortfin mako sharks *Isurus oxyrinchus* (Lamnidae)^14^ to estimate field routine metabolic rates (RMR), total daily energy expenditure (TDE), and feeding requirements of white sharks at a NZ fur seal colony at the Neptune Islands, South Australia.

## Results

**Figure 1:**
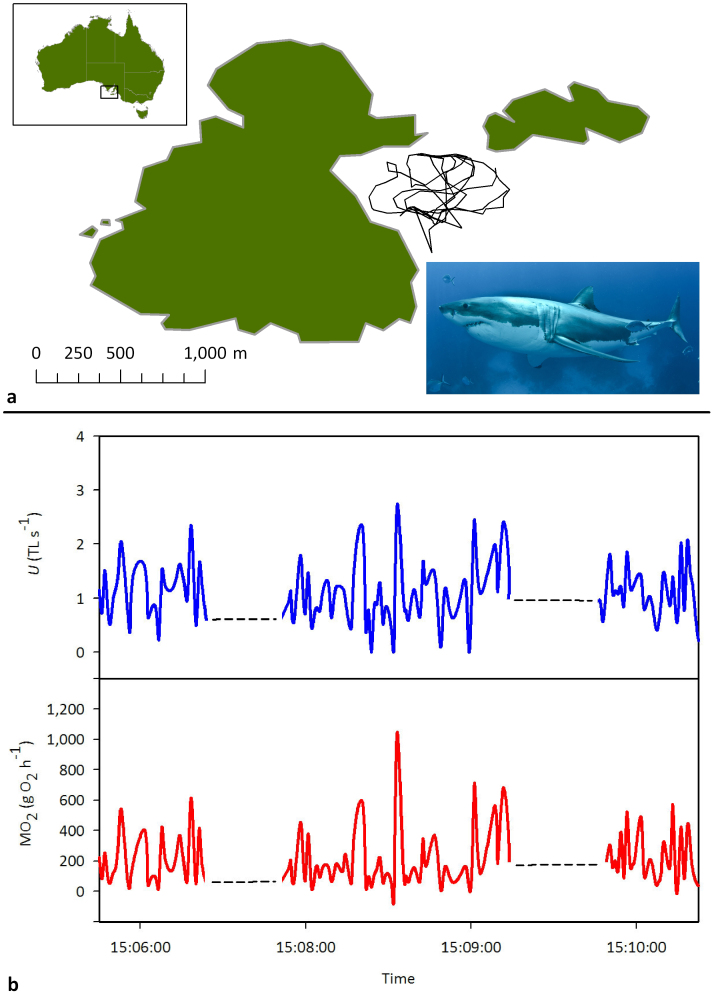
Movements, swimming speeds and metabolic rates of a white shark. (a) 3.5 h track from a 3.5 m male white shark at the Neptune Islands fur seal colony, Australia, determined by a radio-acoustic positioning system. Inset. a white shark *Carcharodon carcharias* at the Neptune Islands. (b) Swimming speeds (*U*, TLs^−1^) were calculated from locations made at ≤5 s intervals in (a) and used to estimate routine metabolic rate (RMR) (MO_2_, gO_2_h^−1^ as per the figure axis label) (see Materials and Methods for details).

Throughout the entire monitoring period, 9,969 swim speed estimates were obtained across all individuals. The distribution of swimming speeds was strongly positively-skewed, so we calculated median swimming speeds as well as mean estimates. The grand mean swimming speed (n = 12) was estimated as 2.91 ± 0.16 m s^−1^ (*U*, 0.81 TL s^−1^), and the median as 2.25 ± 0.14 m s^−1^ (*U*, 0.62 TL s^−1^) [Table 1]. From the mean swimming speed, we estimate the field RMR as 723 mg O_2_ kg^−1^ h^−1^ or a TDE of 28.2 MJ (daily ration of 1.5–1.9% wet body weight d^−1^) for 428 kg sharks (the average from our study) to maintain energy balance [Fig. 1(b) shows absolute RMR and *U* for a 3.5 m (388 kg) shark]. This equates to the consumption of 0.3 weaned NZ fur seal pups (mean wt. 14.6 kg) d^−1^, or 1.0 silver seabream (*Chrysophrys auratus*) (mean wt. 4.5 kg) d^−1^. From the median swimming speed, we would estimate the field RMR as 567 mg O_2_ kg^−1^ h^−1^ or a TDE of 22.1 MJ (daily ration of 1.2–1.5% wet body weight d^−1^). This equates to the consumption of 0.2 weaned NZ fur seal pups d^−1^, or 0.8 silver seabream d^−1^.

## Discussion

Our estimate of total daily energy expenditure (TDE) suggests white sharks feed far more frequently than previously estimated^12^ and does not support the proposal that white sharks could survive at energy balance on 30 kg of marine mammal blubber for 1.5 months (44.1 d). Indeed, the mass-specific MR estimated by Refs. 12 for a 943 kg white shark was more than 12-times lower than our estimate for smaller (428 ± 61 kg, mean ± s.e.m., n = 12) sharks (60 versus 723 mg O_2_ kg^−1^ h^−1^). Given that absolute MR scales with body size with an exponent of ~0.8 for most fish including sharks^15^,^16^, it is unsurprising that our mass-specific MR estimate is higher than that of a much larger animal. However, if the measurements of SMR in ~30 kg sharks^13^ and our measurements of MR in 428 kg sharks are scaled upwards using a mass exponent of 0.79 (Ref. 13), there is strong agreement in absolute MR estimated by Refs. 12 and that measured by Refs. 13 (56.6 versus 55.1 g O_2_ h^−1^ for 943 kg sharks), whereas our estimate of absolute MR (161.8 g O_2_ h^−1^ for 943 kg sharks) is about three times higher. This suggests that, whereas we have estimated metabolic rate in actively swimming animals (RMR), Ref. 12 is likely to have estimated MR approximated by rest (SMR). Our estimated daily ration of 1.5–1.8% wbw d^−1^ is highly comparable to the mean ration (estimated directly from the amount of food eaten) for captive YOY white sharks^17^ (1.2% wbw d^−1^), after scaling for differences in body mass between the YOY and adult white sharks. Furthermore, our daily ration is comparable to that estimated for free-ranging mako sharks^18^ (2.3–2.8% wbw d^−1^), after scaling for differences in body mass between the mako and white sharks.

The new estimate of white shark RMR has implications for assessing the likely feeding frequency of this species. Using our estimate of RMR, 30 kg of blubber (27.9 MJ kg^−1^) would provide a 943 kg (the weight of the shark examined by Ref. 12) white shark with sufficient energy for approximately 11.6 days, which is about four times less than that calculated by Ref. 12 [Table 1]. The winter–spring water temperature at the Neptune Islands, where we recorded the swimming speeds of white sharks, is 15.35 ± 0.86°C (mean ± s.d.). This is very similar to that recorded by Refs. 12 (14.7–16.7°C) during their measurement of MR, and as such cannot in itself account for the high RMR estimated. However, our RMR estimate takes into account the high levels of activity needed for a white shark to ‘patrol’ a seal colony (e.g. 2.9 ± 0.2 m s^−1^, grand mean ± s.e.m, n = 12; 0.81 TL s^−1^), including burst speeds up to 10 m s^−1^ [~2.85 TL s^−1^ for a 3.5 m shark, Fig. 1(b)]. When a median value of swimming speed is used (2.25 ± 0.14 m s^−1^, grand mean ± s.e.m, n = 12; 0.62 TL s^−1^), we get a RMR estimate of 567 mg O_2_ kg^−1^ h^−1^ (absolute RMR 67.9 g O_2_ h^−1^), which is comparable to previous estimates of RMR for the related shortfin mako shark^14^,^19^ (absolute RMR 41.2–44.2 g O_2_ h^−1^, scaled upwards to the mean white shark weight for this study (428 kg) using a mass exponent of 0.79). However, after scaling up to 943 kg using an exponent of 0.79, our absolute RMR calculated from the median value of swimming speed (126.8 g O_2_ h^−1^) is still more than two times that for Ref. 12 [Table 1]. Even at this median RMR value, TDE is equivalent to a daily ration of 1.2–1.4% wbw d^−1^ and 30 kg of blubber providing a 943 kg white shark 14.8 days energy, which is about three times less than that calculated by Ref. 12 [Table 1].

Given their high metabolic rates, white sharks may target seal colonies to predate on seasonally abundant and more vulnerable weaned pups^20^, rather than adult seals or patchily-distributed fish. Silver seabream is a common teleost prey of Australian white sharks^21^, and while the energy density of both prey items are similar (9.4 MJ kg^−1^ and 8.8 MJ Kg^−1^ for weaned seal pups and silver seabream, respectively), the smaller mean size of silver seabream would necessitate at least one (1.0) successful predation event per day to maintain energy balance, compared to less than one (0.3) if targeting weaned seal pups. However, to contribute any energy toward growth and reproduction, they would need to eat more than one silver seabream per day, but would be in positive energy balance if predating on seal pups every third day. Patchily-distributed reef-associated prey such as *C. auratus* have been described as ‘less-visitable’ for white sharks^22^ given the prey's ability to disperse and shelter among complex habitat. Hence, there may be a distinct energetic advantage in targeting one prey item every few days in a predictable (revisitable) habitat such as a seal colony^14^, compared to pursuing and capturing more than one prey item every day in a less-visitable patch (i.e. silver seabream aggregation). During the summer–autumn periods when the weaned pups are not present, white sharks are less common at the Neptune Islands, and during these periods sharks have been tracked moving away from the Neptune Islands to areas where large finfish aggregations (including species such as silver seabream) occur^21^. This movement is accompanied by a shift in search pattern from that approximating Brownian motion at the seal colony (predicted behaviour when prey is abundant) to movement well approximated by a specialized random walk known as a Lévy flight, predicted when foraging for sparsely-distributed prey in more open shelf and pelagic environments^22^. This indicates that feeding on finfish aggregations may be more efficient than foraging for adult seals that are less vulnerable to predation than juveniles^20^.

Our study suggests that due to high metabolic rates, white sharks need to feed more regularly than has been previously assumed^12^,^23^,^24^. Given direct observations of feeding frequency are generally not possible for apex marine predators and that the majority of information available is inferred from behavioural information, field-energetic approaches such as that used in this study may help to answer key ecological questions for a broad suite of such taxa, the populations of which are currently under immense pressure from human exploitation^25^. As an example, our approach could provide a tool for examining the ecological role of mesopredator release through removal of large sharks, such as white sharks. This is a very topical and contentious area of ecological research where further empirical evidence is needed^26^.

## Methods

Twelve white sharks *Carcharodon carcharias* (estimated total length (TL) range: 2.8 – 4.5 m, mean ± s.e.m.: 3.6 ± 0.2; estimated wet body weight (wbw) range: 195 – 839 kg, mean ± s.e.m.: 427.5 ± 60.6 kg) were tagged externally with acoustic depth transmitters (model V16P-5H, Vemco, Halifax, Nova Scotia) at the Neptune Islands, Australia between December 2009 and September 2011. Tagging was carried out under South Australian (SA) Department of Environment, Water and Natural Resources permits M25738 and M25738-2, SA Department of Primary Industries and Resources exemption 9902364 and Flinders University Animal Ethics Committee approval E287. The three-dimensional positions (latitude, longitude and depth) of tagged sharks were triangulated for up to 19 d [for example see Fig. 1(a)] using a radio-acoustic positioning system (Vemco, Halifax, Nova Scotia. Model VRAP), which covered 0.052 km^2^. Swimming speed (m s^−1^) was calculated using consecutive location estimates (≤5 s apart). Above 10 m s^−1^, cavitation limits swimming speeds^27^. As such only swimming speeds below 10 m s^−1^ were used (~10,000 speeds representing 82% of data) to calculate a grand mean swimming speed (m s^−1^) for the 12 sharks. This single value was then converted to *U* (TL s^−1^) using the mean TL.

To estimate field RMR we modified the relationship for oxygen consumption rate (MO_2_, mg O_2_ kg^−1^ hr^−1^) and swim speed (*U*, TL s^−1^) determined directly for a shortfin mako shark *Isurus oxyrinchus*^14^. 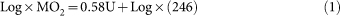
where by the Log value in the intercept 246 in Eq (1) represents the standard (equivalent to resting in an obligate ram-ventilator) MR (SMR, mg O_2_ kg^−1^ hr^−1^) calculated during the transport of captive YOY white sharks^13^, the slope 0.58 Eq (1) represents that determined for a shortfin mako shark, and *U* in Eq (1) is the value calculated from our swim speed estimates (TL s^−1^).

Total daily energy expenditure (TDE, MJ) was calculated from field RMR using an oxycalorific coefficient of 13.55 kJ g^−1^ O_2_ (Ref. 28). To determine the number of weaned NZ fur seal pups needed to be consumed at this TDE to maintain energy balance and the associated daily ration (% wbw d^−1^; calculated as per Ref. 18) we used an energy content value (9.4 MJ kg^−1^) based on that for closely-related Antarctic fur seal pups (*Arctocephalus gazella*)^29^, with a mean weaned NZ fur seal pup weight of 14.6 kg (Ref. 11) and an assimilation value of 73% (Ref. 30). This was also undertaken for a dominant teleost prey of white sharks throughout their Australian range^21^, the silver seabream *Chrysophrys auratus* (estimated mean weight 4.5 kg; 8.8 MJ kg^−1^; Ref. 31). The number of days 30 kg of whale blubber (27.9 MJ kg^−1^) as per Ref. 12 would maintain energy balance at our calculated TDE was also estimated, after scaling up to 943 kg (the weight of the single shark from the study by Ref. 12) using an exponent of 0.79 (Ref. 13).

## Author Contributions

J.M.S. conceived the study; C.H. tagged sharks and set up the tracking system with J.M.S.; J.M.S. and N.L.P. and C.H. proposed and performed the metabolic and spatial analyses, respectively; All authors co-wrote the manuscript.

## Figures and Tables

**Table 1:** Summary of white shark metabolic rate (MR) estimates and the implications for prey intake requirements. Body mass for our study and that of Ref. 13 are given as mean ± s.e.m. All other values for our study are means (grand mean ± s.e.m.) and values in parentheses are medians (grand mean ± s.e.m.). Ref. 13 estimated the range of swimming speeds from video recordings of the sharks swimming in a transport tank. Ref. 12 estimated the shark's swim speed by proxy; the tracking ship's course was stated to approximate that of the fish, giving an over-the-ground speed of 3.2 km h^−1^. Absolute MR and duration of energy balance from 30 kg of blubber was estimated for this study by scaling up to 943 kg using an exponent of 0.79. Ref. 12 used an energy value for blubber of 27.9 MJ kg^−1^

Study	No. individuals	Body mass (kg)	Swimming speed (m s^−1^)	Estimated mass-specific MR (mg O_2_ kg^−1^ h^−1^)	Estimated absolute MR (g O_2_ h^−1^)	Duration (days) that shark is in energy balance from 30 kg blubber
Ref. 12	1	943	0.9	60.0	56.6	44.1
Ref. 13	4	29 ± 2	0.58 – 0.81	246.0	55.1	—
This study	12	428 ± 61	2.9 ± 0.2 (2.3 ± 0.1)	723.0 (566.5)	161.8 (126.8)	11.6 (14.8)
